# Investigation of Complaints against Panel Doctors

**Published:** 1913-06-28

**Authors:** 


### NATIONAL INSURANCE ACT DEPARTMENT.

### LVIII.—Investigation of Complaints Against Panel Doctors.

The medical profession as a body has now acquiesced in the arrangements for the administration of medical benefit under the National Insurance Act, and those doctors who have undertaken service on the panels have apparently found the conditions not so unpleasant or unworkable as had been anticipated, for open expression of discontent is not now so conspicuous as it was. In this connection it is noteworthy, that the Scottish Medical Council, whose object was to oppose the Act, has been dissolved.

While it is gratifying to learn that the doctors as a whole are not so much opposed to the conditions of service on the panels, it is not so pleasant to record that some very loud complaints have recently been made as to the way in which certain panel doctors are carrying out their duties. Names have so far not been publicly divulged, but statements of complaint have been made by well-known approved society officials and by members of Insurance Committees, and little room is left for doubt that there are valid grounds for some of the complaints alluded to. It is more than probable, of course, that the complaints as they have appeared in certain journals have been unduly magnified, and in calling attention to them it is important that we should state that no suggestion is made that panel doctors as a whole, or any considerable proportion of them, are unworthy of their appointments. At the same time the existence of any ground of complaint is unsatisfactory, and it is to be hoped that steps will be taken immediately for its elimination.

### Limitation of Number of Patients.

The complaints against panel doctors are of two classes. In the first place, it has been stated that insured persons have been unable to obtain the treatment to which they are entitled; and, secondly, that doctors are aiding malingerers by lenient practice in regard to the issue of medical certificates.

Complaints as to inadequate attention have not been numerous considering the far-reaching character of the Act and the complexity of its provisions; and it may be that the Insurance Committees responsible for the administration of medical benefit are in such cases as much to blame as the doctor for allowing him to enrol too many insured persons on his list of patients. The remedy for this state of affairs is in the hands of the Insurance Committees, and to prevent the misuse of public funds it is imperative that they should limit the number of patients that may be allotted to any individual practitioner.

### The Supply of Medical Certificates.

Each doctor who has placed his name on a panel has signed an agreement undertaking, amongst other things, to furnish free of charge such certificates as may be required to enable the insured persons on his list to claim sickness benefit from their approved societies. These certificates are required as evidence of the insured person's incapacity for work occasioned by some specific disease or by bodily or mental disablement, and are taken by the societies as *prima facie* evidence of the right of the member to declare on or to continue on the funds of the society as the case may be. The complaints to which we have alluded allege the too free issue of the certificates in question by the doctors concerned, resulting in the admission of claims for benefit by the societies which would not have been possible if the certificates had not been obtained.

The approved societies are now asking for, and apparently hope to obtain, an explicit statement on the certificates as to the nature of the illness or disablement from which the insured person is suffering. The forms of certificate supplied to practitioners were drafted from the outset in most cases to disclose the cause of incapacity for work, but in a large proportion of cases the space has either been left blank or has been filled with the vague words "by illness." The approved societies have been faced in consequence with the disagreeable alternative of risking unpopularity by declining payment of benefit until the certificate has been returned fully completed, or of paying the claims without full particulars which would enable them to supervise the payment, particularly in regard to the duration of illness.

There are other reasons beyond the mere desire for information which make societies wish that the certificates should set forth clearly the nature of the illness or disablement. For instance, the incapacity of a member, which is occasioned by an accident or by an industrial disease, on account of which he is entitled to claim compensation, does not entitle him to benefit from his society, or only for such proportion of the benefit as is required to make up the compensation receivable to the amount payable under the Insurance Act. In these circumstances the societies have a right to expect the assistance of the doctor who furnishes a certificate to protect them against making payments where no valid claim could be substantiated if full information were divulged. Again, most societies have drawn up rules designed to protect their funds against claims where the incapacity of the member results from his personal misconduct or bad habits, and the statement of the nature of the illness is required if such rules are to be enforced. It is argued, on the other hand, by the doctors that a statement of the nature of the illness will frequently be useless in the hands of the local officials of approved societies who have to deal with payment of benefits, and to that extent the extra writing would be so much waste labour. It is further contended that a medical certificate is a confidential document, and that as the doctors are not in the direct employ of the approved societies they cannot divulge the nature of the illness to them. This difficulty has been overcome by the issue of certificates addressed to the insured person himself, and it is then left to the discretion of the member whether he makes use of the form or not.

Certificates to be Used as Required.

A much more serious complaint has been made, however, to the effect that signed certificates have been furnished to insured persons, to be filled in as regards dates and so forth, and made use of when required. An instance of this has come under our personal observation, and another case of a similar description was cited at the Conference of the Ancient Order of Druids held last week at Cardiff. In both cases it is presumably the intention to lodge formal complaints with the Insurance Commissioners, and these will in due course be investigated.

In this connection it may be pointed out that although the National Insurance Act confers the right on any duly qualified medical practitioner of being included on the list of panel doctors, should he desire to be so included, it also gives the right to the Insurance Commissioners to remove his name from the list if, after inquiry in prescribed form, they are satisfied that his continuance in the list would be prejudicial to the efficiency of the medical service of the insured [Section 15 (2) (*b*)].

Commissioners' Regulations as to Inquiries.

The Commissioners have issued provisional regulations, dated June 10 last, for regulating the procedure in inquiries as to the efficiency of medical practitioners.

Briefly stated, the regulations provide that where complaint is made to the Insurance Commissioners by an Insurance Committee or by a Local Medical Committee, the Commissioners must order an inquiry to be held. If the complaint be made by another person or body (including an approved society), an inquiry may or may not be instituted, according to the discretion of the Commissioners. The Inquiry Committee shall consist of two practitioners and a barrister-at-law or solicitor, in actual practice, and the formalities are sufficiently indicated when it is stated that the first schedule contains no fewer than eleven forms for use in giving notice to various parties prior to the inquiry being held.

Witnesses may be heard at the inquiry on behalf of either party, and all witnesses (including the parties) will be subject to examination and cross-examination as nearly as may be as if they were witnesses in an ordinary action. In view of the publication of the complaints to which we have alluded, it is satisfactory to learn that machinery is now in existence which will enable them to be sifted and analysed. At the same time we would express the hope that the machinery will only have to be tested on rare occasions.

**Answers to Correspondents.**

NOTE.—Questions and Correspondence on all doubtful points are invited, and should be addressed to the Editor, The Hospital, 28, 29 Southampton Street, Strand, London, W.C., and marked "Insurance."

